# Prevalence of Maternal Anemia in Southern Jordan: Findings from a Cross-Sectional Study and 5-Year Review

**DOI:** 10.3390/healthcare12242495

**Published:** 2024-12-10

**Authors:** Ahlam M. Al-Kharabsheh, Israa F. Alahmad, Malak M. Al-Tamimi, Seham M. Abufraijeh, Nedal Alnawaiseh

**Affiliations:** 1 Department of Obstetrics and Gynecology, Faculty of Medicine, Mutah University, Alkarak 61710, Jordan; sehammahmoud@mutah.edu.jo; 2 Faculty of Medicine, Mutah University, Alkarak 61710, Jordan; 3 Department of Public Health, Faculty of Medicine, Mutah University, Alkarak 61710, Jordan; nawayseh@gmail.com

**Keywords:** anemia, pregnancy, Jordan, antenatal care, maternal health, adolescent pregnancy

## Abstract

**Background:** The literature underrepresents maternal anemia in southern Jordan, and national studies often have small sample sizes. According to the 2019 Jordan National Micronutrient Survey, maternal anemia in Jordan accounted for 19.1% of cases, with the majority experiencing mild anemia (17.1%). Among the 29 pregnant women recruited from the southern region, 56.5% had anemia, of which 34.8% had mild and 21.7% had moderate anemia. The current study provides an update on the prevalence of anemia during pregnancy in southern Jordan. **Methods:** This cross-sectional study was conducted at a government referral hospital in southern Jordan. Pregnant women who visited outpatient clinics were included in the study. Data were collected through face-to-face interviews, and blood samples were collected to measure hemoglobin levels. Descriptive analyses of continuous and categorical variables were performed. Maternal anemia risk factors were assessed using the chi-square test, Fisher’s exact test, and multivariate logistic regression analysis. Statistical significance was defined at *p* < 0.05. **Results:** Of the 474 pregnant women who agreed to participate, 36.8% had anemia. Education, income, diet (number of meals and meat consumption), regularity of antenatal care, and supplement use were factors that significantly affected hemoglobin levels (*p* < 0.05). The adolescent pregnancy rate was 5.3%, and 48% had mild to moderate anemia. **Conclusions:** Maternal anemia rates in southern Jordan remained high, despite a slow decline. Reducing this burden requires improved access to healthcare and health education, particularly for rural residents who are at greater risk of disease.

## 1. Introduction

Anemia during pregnancy is defined by the Centers for Disease Control and Prevention as hemoglobin levels< 11 g/dL in the first and third trimesters and <10.5 g/dL in the second trimester [1] and is primarily caused by iron deficiency in over 50–70% of pregnant women with anemia, globally [2]. This condition is associated with serious health risks during pregnancy and is widely reported. A 2023 review and meta-analysis of 148 studies [3] investigated the relationship between maternal anemia and adverse outcomes. Associations were found between anemia defined as hemoglobin levels less than 11 g/dL and low birth weight, preterm birth, small for gestational age (SGA) fetuses, stillbirth, perinatal mortality, and neonatal mortality [3].

Regarding maternal outcomes, Young et al. [3] have reported that lower hemoglobin levels were associated with postpartum hemorrhage, the need for blood transfusion, the development of pre-eclampsia, and the risk of antenatal depression. The overall association between anemia (≤11 g/dL) and maternal mortality was non-significant; however, a significant association emerged at lower hemoglobin thresholds as follows: ≤10 g/dL (OR 2.87, 95% CI 1.08–7.67) and ≤9 g/dL (OR 4.83, 95% CI 2.17–10.74) [3].

Because of the potential consequences of anemia in pregnancy, several global calls started to work on resolving this critical public issue. A systematic review conducted in 2013 revealed a downward trend in the prevalence of anemia among pregnant women worldwide, from 43% to 38% [2]. The Middle East and North Africa (MENA) region had one of the highest prevalence rates at 31%. The severe anemia prevalence in MENA was 0.4% [2]. A 2019 World Health Organization (WHO) update indicated a global prevalence of 36.5%, with the rate in Jordan at 33.7% and a mean hemoglobin level of 11.5 g/dL [4].

The Global Burden of The Disease study identified maternal malnutrition as a major risk factor for disability-adjusted life years, with iron deficiency being the leading cause for women aged 10–24 years [5,6]. The WHO has targeted a 50% reduction in anemia among women of reproductive age by 2025 [7], which is an important step in improving maternal health and reaching Millennium Development Goals [8].

The 2017–2018 Jordan Population and Family Health Survey (JPFHS) reported an anemia prevalence of 43% among women aged 15–49 years, with rates of 35% in Madaba and 49% in Ma’an [9]. Only 49% of pregnant women received adequate iron supplementation, and 20% received no iron during their last pregnancy [9].

The 2019 Jordan National Micronutrient and Nutrition Survey (JNMNS) [10] found that 56.5% of pregnant women in southern Jordan were anemic, compared to 26.5% in the north and 13.3% in the central areas. Anemia severity ranged from mild to moderate; 34.8% of the southern women had mild anemia, while 21.7% had moderate anemia. No cases of severe anemia were reported.

The current study aims to provide updated information about maternal anemia in south Jordan, a region where the existing literature is both limited and poorly documented. Our hypothesis is that the prevalence of maternal anemia has decreased since the 2019 JNMNS [10] reported that pregnant women in the south were 2–3 times more likely to be anemic compared with women living in the central and northern parts of the country. In order to investigate this hypothesis, we conducted a cross-sectional study that was intended to establish the current prevalence of anemia in pregnancy, comparing such status to the JNMNS results and ascertaining the influence of sociodemographic, dietary, and obstetric parameters on the appearance of anemia.

Preliminary data expanded our focus on the adolescent pregnancy rate and specific risk factors associated with maternal anemia within this cohort.

## 2. Materials and Methods

### 2.1. Study Area, Population, and Design

The Jordan Department of Statistics (2023) estimates the population of the southern region to be 916,000, with 41.5% residing in Al-Karak, a major governorate situated 124 km south of Amman [11]. Al-Karak and the nearby governorates, like Al Tafila, face socioeconomic challenges, particularly limited access to comprehensive healthcare services. Al Karak Governmental Hospital, the main referral center for southern Jordan, served as the study site.

A cross-sectional survey was conducted between February 2024 and July 2024, utilizing a structured questionnaire in outpatient clinics.

### 2.2. Inclusion and Exclusion Criteria and Study Sampling

Pregnant women visiting outpatient clinics for routine antenatal care who consented were included in the study.

Women with multiple pregnancies were excluded to maintain homogeneity and minimize bias, as these pregnancies are associated with an increased maternal anemia risk. Women with conditions affecting hemoglobin levels, such as hemoglobinopathies (thalassemia or sickle cell anemia) or chronic renal disease, were also excluded.

The sample size was calculated based on the reported 56.5% anemia prevalence among pregnant women in southern Jordan (JNMNS) [10]. The sample size was calculated via the following formula: n = (Z^2^ × p × (1 − p))/E^2^.

Here,

- n = required sample size;
- Z = desired confidence level (for a 95% confidence interval, Z was typically 1.96);
- p = prevalence of anemia from previous studies (56.5%);
- 1 − p = proportion of the population without anemia;
- E = margin of error (0.05).

The minimum sample size required for the study was 370. We increased the sample by 100 participants to account for potential dropouts and enhance the statistical power.

### 2.3. Data Collection

Sociodemographic data were collected using structured questionnaires administered via face-to-face interviews with the women at the clinic. During this phase, and to secure confidentiality and privacy, only those qualified and willingly consenting to participate in the study were interviewed alone in private settings. Places were chosen in order to ensure that respondents felt safe and comfortable. In this way, we encouraged open and honest communication while safeguarding their personal information.

Collected data included age, education, employment status, family size, income, residence, and dietary habits, such as frequency of meals, meat consumption, tea consumption (an iron absorption inhibitor), and smoking status. Obstetric data included gestational age (calculated from the last menstrual cycle), parity, interpregnancy interval, prenatal check-up attendance, and supplement intake (folic acid, iron, multivitamins). Additionally, the medical histories, including those of diabetes, hypertension, hemorrhoids, and peptic ulcers, were recorded.

### 2.4. Study Variables

#### 2.4.1. Explanatory Variables

Sociodemographic and obstetric characteristics are as follows: age groups (<20, 20–25, 26–34, and 35–45 years), self-reported preconception weight and height (used to calculate body mass index [BMI]). BMI categories were defined according to the WHO classification as follows: underweight (BMI ≤ 18.5 kg/m^2^), normal weight (BMI 18.5–24.9 kg/m^2^), overweight (BMI 25–29.9 kg/m^2^), obese class I (BMI 30–34.9 kg/m^2^), obese class II (BMI 35–39.9 kg/m^2^), and obese class III (BMI ≥ 40 kg/m^2^) [12]. Parity was classified into primigravida (no delivery), multiparous (1–4 and ≥5 deliveries), gestational age (first, second, and third trimester), interpregnancy interval (<1 year, 1–2 years, and >2 years), place of residence, level of education (elementary, high school, and university), employment status (yes/no), household income (<200, 200–500, 500–1000, >1000 Jordanian dinars [JOD]), active smoking status (yes/no), husband (passive) smoking status (yes/no), and family size (2, 3, 4, and >5 households).

Second, dietary characteristics and nutritional supplements included the number of meals per day (1, 2, 3, ≥4 times), daily tea intake (none, once, and multiple times), meat consumption (daily, twice per week, once per week, and 1–3 times per month), folic acid consumption (yes/no), iron supplement consumption (yes/no), and multivitamin consumption (yes/no).

Third, medical conditions included hemorrhoids (yes/no), peptic ulcers (yes/no), and other comorbidities such as diabetes and hypertension (yes/no).

#### 2.4.2. Outcome Variable

Anemia was defined, according to WHO criteria, as hemoglobin< 11.0 g/dL [13]. Anemia severity was categorized as mild (9.0–10.9 g/dL), moderate (7.0–8.9 g/dL), and severe (<7.0 g/dL) [13]. Venous blood samples were collected after patient consent and analyzed to determine hemoglobin levels.

#### 2.4.3. Definitions of Variables

Our hospital adopted the WHO 2016 antenatal care guidelines [14], recommending a minimum of eight contacts during pregnancy to improve care quality and reduce obstetric complications and stillbirths.

The poverty line was defined as an income below 2.15 US dollars (USD) per day per individual, equivalent to JOD 1.52 [15]. Household income was assessed in relation to this standard to provide a context for socioeconomic status (SES).

The interpregnancy interval (IPI) is the time between the end of one pregnancy and the start of the next conception. A short IPI was defined as <24 months [16].

Pregnancy during adolescence (10–19 years) was classified as adolescent pregnancy [17].

### 2.5. Statistical Analysis

Data were analyzed using the Statistical Package for the Social Sciences software (SPSS, version 25) in assessing the prevalence of anemia. The study used descriptive statistics to summarize the sociodemographic characteristics as follows: for continuous variables, means and standard deviations were calculated; for categorical variables, the findings were summarized using frequencies and percentages. Bivariate analyses of the relationship between anemia and its risk factors were performed with chi-square tests for the categorical variables and *t*-tests or analysis of variance for continuous variables. All the variables that showed a *p*-value of less than 0.05 in the bivariate analysis were selected for further evaluation using the multivariate logistic regression method.

Multivariate logistic regression was used to identify independent predictors of anemia, while controlling confounding factors by including them in the model. The assumptions of the model were tested through Hosmer and Lemeshow’s goodness of fit test.

Multicollinearity between the predictor variables was checked using the variance inflation factor, and all the values were below the acceptable threshold, indicating no multi-collinearity. Hence, the variables were included in the model.

Adjusted odds ratios (AOR) and 95% confidence intervals (CI) were calculated. Statistical significance was set at *p* < 0.05. The dependent variable was anemia status, dichotomized into anemic and non-anemic. The independent variables were categorized; only those with significant associations from the bivariate analysis were included in the multivariate model.

### 2.6. Research Ethical Approval

This study complied with the Declaration of Helsinki principles. Informed consent was obtained from all participants, with legal guardians providing consent for those under 19 years old. The study was approved by the Institutional Review Board of Mutah University (reference number: 1472024). Participants’ confidentiality was ensured throughout the data collection process, and data dissemination protected participants’ anonymity.

## 3. Results

### 3.1. Anemia Prevalence and Severity

**Figure 1:**
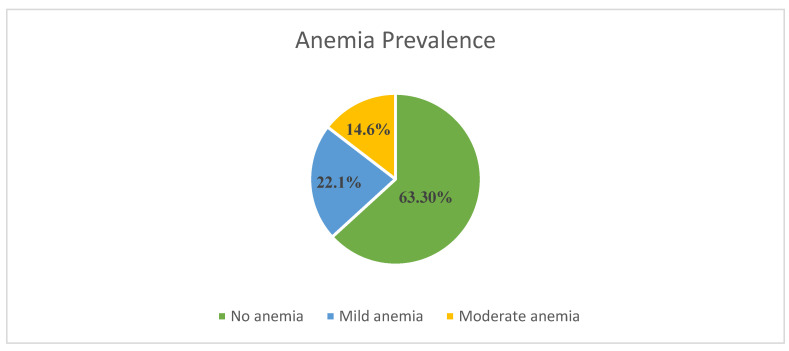
Prevalence and degrees of anemia among the respondents.

The prevalence and severity of anemia among the respondents are shown in Figure 1. This study enrolled 474 pregnant women attending antenatal clinics at Al Karak Hospital, with 174 (36.8%) diagnosed with anemia. The mean hemoglobin concentration was 11.42 g/dL (±1.43), with a median of 11.6 g/dL. Most participants (63.3%) who had hemoglobin levels ≥ 11 g/dL, 105 (22.1%) had mild anemia, and 69 (14.6%) moderate anemia. No severe cases were reported.

### 3.2. Sociodemographic, Medical, and Diet-Related Characteristics

The Sociodemographic, medical, and diet-related characteristics of respondents are illustrated in Table 1. The majority (81.2%) were from the Al Karak governorate, with minor representations from the districts and other governorates. The prevalence of maternal anemia, in relation to origin, was noted by location as follows: 34.8% of women from Al Karak were anemic, 47.6% from Al Qatranah, 61.5% from Ghor Al Safi, and 31% from Al Tafilah.

The mean age was 29.04 ± 6.405 years, with a median of 30 years, and 47.5% were aged 26–34 years, where anemia incidence was highest (36%). The median BMI was 24.6 kg/m^2^; 28.3% were overweight and 21.5% obese. Anemia was more common in those within the underweight category (BMI < 18.5 kg/m^2^) and in women with class I obesity (BMI 30–34.9 kg/m^2^), with rates of 42.6% and 44.1%, respectively, compared to their counterparts.

Regarding education, 19.4% completed elementary school, 40.5% secondary school, and 40.1% university. The highest anemia rate (57.7%) was found among those with elementary education. Most participants (79.5%) were unemployed, and 95% of these women had anemia.

Among those with a household income below JOD 200, 55.3% were anemic. Smoking prevalence was low at 3.6%.

### 3.3. Obstetric Characteristics

The obstetric characteristics of the participants are shown in Table 2. Distribution of parity showed that 53.4% had one to four children, 30.1% were primiparous women, and 16.5% had five or more offspring. Anemia was especially common among multiparous women; affecting 39.5% of those who had one to four children and 37% of those with five or more, compared to 31% of primiparous women.

Among the multiparous participants (n = 331), around 60% reported a short IPI and 61.9% had a previous cesarean section. Women with a shorter interpregnancy interval (given that short IPI is defined as less than 2 years) were more likely to have anemia than those with intervals longer than 2 years. The overall percentage of anemia among women with a short interpregnancy interval was 41.9%, in contrast to 35.3% among women with longer pregnancy intervals.

Among women, 12.7%, 36.5%, and 50.8% were in their first, second, and third trimesters, respectively. The mean gestational age was 25.24 ± 7.87 weeks with a median of 28 weeks. Forty-one percent of women in their third trimester of pregnancy were found to have maternal anemia. The majority of anemia cases at different gestational ages were classified as mild anemia, which constitutes 88.5% of all anemia cases (154 out of 174).

A total of 75.1% (356/474) of the participants maintained regular antenatal care, among whom 69.1% (246 out of 356) displayed normal hemoglobin levels, 28.4% (101 out of 356) displayed mild anemia, and 2.5% (9 out 356) presented with moderate anemia.

Nutritional supplement intake was high, with 93.9% of participants consuming folic acid, 64.1% consuming iron, and 62% consuming multivitamins. Compared with their counterparts, women who took supplements had lower rates of anemia. The incidence rates of maternal anemia among those who did not consume folic acid, iron, or multivitamins were 72%, 44%, and 52%, respectively.

### 3.4. Sociodemographic, Economic, Medical, and Diet-Related Predictors of Anemia

The sociodemographic, economic, medical, and diet-related predictors of anemia among the respondents are presented in Table 3. In the bivariate analysis, six variables with a *p*-value below 0.05 were identified as significant and included in the multivariate regression model. These variables were residence, education level, employment status, household income, meals per day, and dietary iron intake (meat). Upon multivariate analysis, most of these factors remained statistically significant, with some variations observed in the categories of two factors, residence and frequency of meat intake.

Pregnant women residing in Ghor Al Safi were significantly more likely to develop anemia, with a 72% increased likelihood compared to those from Al Karak (OR = 1.722; 95% CI: 1.244, 2.384; *p* = 0.014). In contrast, for women from Al Qatranah and Al Tafilah, the associations were not statistically significant. However, the likelihood of anemia was slightly increased for women from Al Qatranah by 28.9% (OR = 1.289; 95% CI: 0.666, 2.496; *p* = 0.531) and notably reduced for those from Al Tafilah by 21.2% (OR = 0.788; 95% CI: 0.370, 1.678; *p* = 0.619).

Regarding the frequency of meat consumption, women who consumed meat daily had a decreased risk of developing anemia, though this was not statistically significant, at just over fifty percent (OR = 0.436; 95% CI: 0.149, 1.279; *p* = 0.138). In contrast, women who consumed meat less frequently, that is, once or twice a week, had a significantly reduced anemia risk (OR = 0.307; 95% CI: 0.168, 0.560; *p* < 0.001 and OR = 0.393; 95% CI: 0.255, 0.608; *p* < 0.001, respectively).

Those with higher levels of education showed a significantly lower anemia development risk. Those who reached secondary education had a 30.6% reduced risk of developing anemia (OR = 0.694; 95% CI: 0.542, 0.889; *p* = 0.007), while those at university level showed a 59% reduced risk of developing anemia (OR = 0.407; 95% CI: 0.298, 0.555; *p* < 0.001).

Employment status was a protective factor, and women who were employed had a 40% lower chance of having anemia compared to unemployed women (OR = 0.603; 95% CI: 0.370, 0.985; *p* = 0.045).

No cases of anemia among women with incomes above JOD 1000 were reported, indicating the most robust protective effect. As income increased, the risk of anemia, though at a lower level, also significantly decreased; the risk was reduced by 72.1% for those earning JOD 501–1000 (OR = 0.279, 95% CI: 0.150, 0.522, *p* < 0.001) and by 66.7% for those earning JOD 200–500 (OR = 0.333, 95% CI: 0.218, 0.508, *p* < 0.001).

Finally, a higher number of meals consumed per day was associated with lower odds of developing anemia, as shown in Table 3.

### 3.5. Obstetric Predictors of Anemia

The obstetric predictors of anemia among the respondents are presented in Table 4. As indicated in Table 2, bivariate analysis showed a significant relationship between the development of anemia during pregnancy and both the attendance at regular antenatal care and the consumption of nutritional supplements, such as folic acid and multivitamins (*p*-values < 0.001), as well as iron (*p*-value = 0.01).

Pregnant women who regularly attended antenatal care sessions had 54% lower odds of having anemia when compared to those with irregular attendance of antenatal check-ups (AOR = 0.457; 95% CI: 0.293, 0.715; *p* = 0.001). Similarly, the multivariate model demonstrated a significantly lower risk of anemia among women who used folic acid and multivitamin supplements. In fact, the risk was reduced by 67% for folic acid (AOR = 0.327; 95% CI: 0.135, 0.794; *p* < 0.05) and 62% for multivitamins (AOR = 0.382; 95% CI: 0.235, 0.621; *p* < 0.001) when compared to women who did not take the supplements.

Nonetheless, the significant association with iron in the bivariate analysis did not remain significant in the multivariate model (AOR = 1.230, 95% CI: 0.749, 2.021, *p* = 0.413), suggesting that its effect is mediated through other factors. Women that received iron had 23% higher odds of being anemic compared to women who did not receive iron; however, this finding is nonsignificant, suggesting the 23% increase in odds is likely due to sampling variability. This unexpected result is contrary to the expected protective effects of iron supplementation, possibly because of confounding variables, differential compliance, or insufficient dosing in this population.

#### 3.5.1. Prevalence and Degree of Anemia Among Pregnant Adolescents

**Figure 2:**
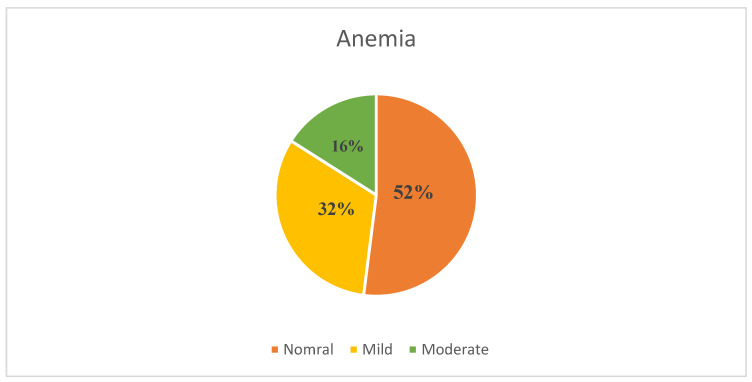
Prevalence and degree of anemia among pregnant adolescents.

The prevalence and degree of anemia among pregnant adolescents is presented in Figure 2. In the cohort, 5.3% (n = 25) of the participants were younger than 20 years of age; none had severe anemia, four (16%) had moderate anemia, eight (32%) had mild anemia, and 52% had normal hemoglobin levels.

#### 3.5.2. Sociodemographic, Diet-Related, and Obstetric Characteristics of the Adolescent Respondents

Table 5 presents the sociodemographic, diet-related, and obstetric characteristics of the adolescent respondents. The mean age of the participants, who were all adolescents (N = 25), was 18.29 ± 0.845 years; 20% were underweight and 60% had attended elementary school. None were employed, while 44% had a family income of below JOD 200. Anemia was more prevalent among those with a BMI < 18.5 kg/m^2^, those from Ghor Al-Safi, and those with a low household income. As previously mentioned, all participants were unemployed, and the prevalence of anemia examined according to employment status was the same as the overall prevalence in this cohort. Women who consumed tea several times daily or ate meat one to three times per month had higher rates of anemia (60% and 53%, respectively). Among the pregnant adolescents, 64% were in the third trimester, and 36% were in their second trimester, with no first-trimester participants. Mild anemia was prevalent across the reported gestational ages, with moderate anemia only observed in the third trimester.

Among this cohort, seventeen (68%) were primigravida and eight were para 1–4. All multiparous women had short interpregnancy intervals shorter than 2 years, 50% of these had a previous history of cesarean section. Anemia was more common among multiparas with intervals of shorter than 1 year, while all the cases with prior cesareans were anemic.

Fifty-six percent of women had regular antenatal visits. Folic acid, iron, and multivitamins use were 80%, 64%, and 52%, respectively. Anemia was more prevalent among those with irregular check-ups (63.6%) and in women not taking folic acid or multivitamins. Notably, 50% of the women who consumed iron were still anemic.

## 4. Discussion

The present study investigated maternal anemia in southern Jordan to explore its prevalence and determinants and compare it with the available evidence from the literature. Although several studies have examined anemia in Jordan, they have focused primarily on the northern area [18,19] or Amman [20,21,22,23]. Others have conducted surveys nationwide, including a study that specifically examined anemia during pregnancy [24,25]. Despite its inclusion in national studies with relatively small sample sizes, the southern region has not been targeted in any previous investigation.

Our study indicated a significant decrease in anemia prevalence among pregnant women compared to a 2012 study [25], where rates were reduced by half, aligning with the WHO 2025 nutritional targets [7]. Based on the WHO categorization regarding the public health significance of anemia [13], our findings show that the prevalence in southern Jordan has decreased from a severe category level [10,25] to a moderate level, as indicated by our reported rate of 36.8%.

Compared to the JNMNS [10], which reported a higher anemia prevalence (56%) based on only 29 pregnant women from the south, our larger sample size provides more representative findings of maternal anemia prevalence in the southern region.

Although our anemia rate remains higher than what was previously reported in northern and central Jordan (26.5% and 13.3%, respectively) [10] and neighboring countries, such as Saudi Arabia (27.3%) and Egypt (26%), it is comparable with that in Syria (33.2%) and Iraq (30.9%) [4].

### 4.1. Socioeconomic and Demographic Determinants

The determinants of the prevalence of anemia in pregnant women include the mother’s age, geographic region, socioeconomic status, conditions of employment, educational attainment, and nutritional intake. All these variables are deeply examined by the available literature. However, their impact on maternal anemia shows substantial variability.

The relationship between maternal age and anemia is particularly inconsistent. According to a 2023 systematic review of the global prevalence of anemia in pregnant women [26], some studies are shown to have found a higher risk of anemia in women aged 35 years or more, while other studies suggested that the women in the age range of 25–33 years had a higher risk. In contrast, our study did not find any significant association between maternal age and the risk of anemia, *p* > 0.05, and that is consistent with other studies from Jordan [10,18].

Education is another controversial factor in the literature. Our results show that higher education is significantly associated with a lower risk of anemia (OR = 0.407; 95% CI: 0.298, 0.555, *p* < 0.001) for those who attained university, possibly because of a better knowledge of the nutritional needs during pregnancy. However, others have contradicted this view while proposing several theories. First, economic factors play a significant role in the relationship between education and anemia [26]. Research indicates that women with limited resources are at a higher risk of anemia regardless of higher education levels, as they face barriers in accessing the essential resources needed to alleviate the condition [26]. Second, prior experiences related to childbirth, particularly among multiparous women, may lead to a decreased prevalence of anemia. This is attributed to enhanced knowledge and improved access to healthcare services [26].

Additionally, geographical context is one of the important determinants of the prevalence of anemia. Women of reproductive age living in rural areas have higher anemia rates than urban-residing women, which is mainly attributed to limited access to nutritional knowledge, health facilities, and medical services [26]. In 2022, Jordan’s Department of Statistics recorded differences in the rural to urban population distribution among different governorates [27]. Our sample comprised women from various geographic areas in the south. The maternal anemia risk was found lower among women in Al Tafila compared to Al Karak, however, without statistical significance; *p* > 0.05. This could be attributed to the fact that 22% of Al Tafila’s population is rural, while in Al Karak, it is 41% [27]. We have also found that women from Ghor Al Safi in the governorate of Al Karak have an increased risk of developing maternal anemia, which warrants specific investigation in this area.

### 4.2. Nutritional, Micronutrient Status and Preventive Measures

The 2017–2018 JNMNS [10] provided important information on micronutrient deficiencies, including anemia, in numerous age groups and Jordanian regions. This survey showed the highest prevalence of iron deficiency at 65.8%, and an anemia prevalence of 37% among non-pregnant women of reproductive age in the southern region, with statistically significant differences observed between the regions. Thus, these findings, theoretically, have placed the women with high preconceptional rates of iron deficiency in this region at a higher risk of IDA in pregnancy, given the increased physiological demands for red blood cell mass expansion and fetal and placental development. Because anemia is a late manifestation of iron deficiency, such women are at risk of progressing from mild to moderate or severe anemia during pregnancy if left untreated.

Our findings indicated that 31.6% of women in the first trimester of pregnancy had anemia, classified as mild or moderate, but with no cases of severe anemia. While specific causes of anemia (iron, folic acid, or vitamin B12) were not explored in our cohort, the high prevalence of iron deficiency in non-pregnant women from the south reported in JNMNS suggests the potential contribution of preexisting nutrient deficiencies to the incidence of anemia during early pregnancy in our sample.

The International Federation of Gynecology and Obstetrics (FIGO) recommends that all women in the reproductive age group should be investigated for iron deficiency and appropriately treated before conception, particularly in those who are planning to become pregnant [28]. Proper screening and treatment are important in reducing risks associated with this micronutrient deficiency and adverse pregnancy outcomes such as abruptio placenta, preeclampsia, preterm labor, low birth weight, SGA fetuses, and postpartum hemorrhage [28]. Furthermore, iron is necessary in fetal and postnatal neurodevelopment, which underlines its role in ensuring maternal and child health [28].

According to the JNMNS [10], serum folate deficiency was significantly (*p* < 0.001) more prevalent among non-pregnant women in the south, reaching 22.3%, compared with those from the northern or central regions. This is possibly due to the success of national efforts to increase folic acid supplement use (93.3%), as shown in our study.

Additionally, our data revealed higher rates of iron and multivitamin supplement use, 64% and 62%, respectively, compared to the JNMNS figures, 56.9% and 29.2%, respectively [10]. The decreased odds of maternal anemia among the folic acid and multivitamin supplement users in our study suggest that supplementation plays a role in reducing anemia prevalence.

Our study also highlights the absence of a significant correlation with iron supplementation, indicating that factors such as multivitamins and folic acid either mediate or obscure its protective effect; these factors remain key predictors for reduced anemia risk. This finding suggests that the cause of anemia in this cohort is likely complex, with other possible causes including folate deficiency or poor diet. Further, adherence to and the dose of iron supplements may influence their effectiveness, which needs further investigation. This lack of association with iron was reported by Al-Mehaisen et al. [18] and they attributed it to the fact that, in Jordan, the standard multivitamin tablets contain iron.

Notably, there were no cases of severe anemia in this study, which was similar to the JNMNS data from 5 years ago [10], though Salahat et al. [25] reported, 12 years ago, a prevalence of 3%. Several factors have probably contributed to this. First, the high percentage of women taking supplements improved hemoglobin levels and prevented severe anemia. Second, 75% of our cohort received regular antenatal care that enabled the early detection of anemia and nutritional deficiencies and intervention through routine blood testing. Third, conducting the study at a referral hospital may have contributed to the reduced occurrence of severe anemia cases.

### 4.3. Non-Supplemental Factors Contributing to Maternal Anemia

The association of higher hemoglobin levels with the use of supplements shows that these may help in maintaining normal levels, although this is also suggested by previous studies [18,29]. However, supplementation alone is not enough to prevent anemia, as 32.5% of our sample who are using iron, 34% of those taking folic acid, and 47% using multivitamins had mild to moderate anemia. This could mean that both knowledge about diet and compliance with supplementation are significant variables to evaluate. The WHO has recommended behavioral strategies regarding this issue to manage and prevent anemia [30].

Additionally, national initiatives aim to improve awareness in women regarding anemia during pregnancy, such as that by Abujilan et al., “Health Information Package Program (HIPP)”, which provides information via videos, WhatsApp messages, and face-to-face interviews [19]. This approach has been associated with higher hemoglobin levels and provides evidence that even regular antenatal care and supplementation may not be able to completely address the non-iron-related reasons for anemia.

Also, the women in our study from a higher socioeconomic background showed significantly higher levels of hemoglobin, reflecting the positive effect of SES on nutrition. This conclusion is further supported by our finding that no cases of anemia were recorded among women whose incomes exceeded JOD 1000. Talin et al. found that women from lower socioeconomic backgrounds are more likely to develop maternal anemia due to limited access to iron-rich foods and proper healthcare services [31].

In terms of the interpregnancy interval influence, the literature shows conflicting evidence concerning IPIs and maternal anemia. While some studies support the “maternal depletion theory” links short IPIs and anemia [32], others show an increased risk with both short and long IPIs [33]. However, other studies, including ours, have found no significant association [18,34], warranting further investigation.

### 4.4. Adolescent Pregnancy: Trends and Implications

Adolescent pregnancies in Jordan have a reported prevalence of 6.2% [35], based on data from a national study named “Neonatal mortality causes and risk factors”, conducted between 2011 and 2012, with regional variations of 6.1% in the north, 6.6% in the central regions, and 4% in the southern regions. It is worth noting that no other studies from Jordan have explored this topic.

According to the 2017–2018 JPFHS [9], 5% of ever-married adolescent women aged 15–19 years had initiated childbearing. Our study revealed that 5.3% of the participants were between the ages of 15 and 19 at the time of the survey.

The association between adolescent pregnancy and maternal anemia remains debated. Salahat et al. reported that 55% of women aged 16–19 years had mild anemia, while 12% had moderate-to-severe anemia [25]. However, other studies suggest that although teenage mothers are at greater risk of preterm delivery, they may not face an increased risk of low birth weight, maternal anemia, or neonatal mortality [36].

During the study period, no adolescents were seen in the first trimester, 36% were in the second, and 64% were in the third trimester. Among pregnant women in both gestational categories, 48% were anemic. In the second trimester, 55% had mild anemia. In the third trimester, 25% had mild anemia, and 25% had moderate anemia.

The previously noted pattern of the progress in maternal anemia from mild to moderate with advancing gestation is highly contributed to the increased physiological demands during pregnancy, coupled with nutritional deficiencies. The above shortcomings could result from social factors, such as low SES, or poor regulation and care during ANC. This is reflected in our adolescent population where only 56% practiced regular ANC, all were unemployed, and the household monthly incomes were less than JOD 500 (USD 705).

The WHO, in its recommendation regarding antenatal care [14], emphasizes the importance of providing timely and evidence-based protocols during ANC for pregnant women and adolescent girls. The aim of these guidelines is to establish a foundation for healthy motherhood. Among the 39 recommendations [14], the focus on nutritional interventions includes the evaluation of various vitamin and mineral supplements for which evidence exists to support their role in improving maternal and perinatal outcomes. Additionally, dietary education can help in mitigating the impacts brought about by undernutrition and overnutrition [14].

WHO specifically recommends iron and folic acid supplementation to prevent maternal anemia, low birth weight, and preterm birth (<34 weeks of gestation) [14]. Supporting this, Mina et al. [36] reported that adequate ANC is a highly cost-effective intervention to reduce adverse perinatal outcomes such as intrauterine growth restriction, preterm birth, perinatal death, and neonatal ICU admission.

Among adolescent pregnant women, the GBD 2019 study [5] has underlined the continued incidence of preterm birth and low birth weight due to child and maternal malnutrition, which contributes massively to disability-adjusted life years across the world. It also highlights the fact that since adolescents are in a state of growth and also due to pregnancy demands, they are at risk of poor birth outcomes that will affect neonatal health, development, and maternal well-being because of nutritional needs.

This evidence underpins global health goals to reduce risks through better antenatal care, nutrition, and policies for adolescent health and education.

Furthermore, adolescent pregnancy has a long-term effect on the education and SES of both mother and child. Health complications and early responsibilities related to motherhood often stand in the way of the mother’s education and economic progress, which may cause cycles of poverty and reduced prospects. A WHO discussion paper on adolescence [37] points out that adolescent pregnancy has far-reaching consequences, and in the same paper, five studies assessing the outcomes of adolescent childbearing in comparison to adult motherhood were critiqued. In all these, it was found that adolescent motherhood is associated with negative socioeconomic conditions and low-earning potential [37]. One important poverty determinant in many countries was the premature termination of education because of adolescent childbearing [37]. Moreover, it was noted that children of teen mothers had lower scores in the area of language development tests, and these mothers reported more behavioral problems in their children [37].

### 4.5. Strengths and Limitations

This study on the largest cohort of pregnant women from southern Jordan provides an accurate estimate of maternal anemia prevalence and thus fills a significant gap in the current literature, as previous studies had limited numbers of participants [10,24,25].

However, there are some limitations to this paper that need to be mentioned. To begin with, it excluded some variables that may affect anemia, such as menstrual history and episodes of bleeding during pregnancy. Likewise, the nationality of the respondents, whether exclusively Jordanians or a mix, including mostly Syrian refugees, was not examined as a variable, even though its impact on the findings had been clearly discussed in the JPFHS and the JNMNs [9,10].

Second, conducting this study in a single referral hospital presents major generalizability challenges. The quality of care provided in referral hospitals is usually higher and may influence estimates of maternal anemia differently compared to settings with limited resources. Given that this study is carried out in only one center and the subjects recruited represent only a portion of pregnant women under medical care, the results may not be representative of the general population of pregnant women in southern Jordan.

Additionally, the research relied on self-reported data on dietary behavior, sociodemographic characteristics, and obstetric histories, which are subject to biases due to recall and social desirability. While this was partly overcome by using structured questionnaires and trained interviewers who conducted the interviews in private settings, the potential for misreporting is inherently unavoidable. Future studies should validate information from medical records or other objective measures.

Furthermore, another major threat to the internal validity was the SES of the participants; 79% of the sample were unemployed, and 87% reported that their monthly household income was below JOD 500, resulting in a lack of variation in SES within the sample. Such homogeneity poses threats to the generalization of the findings to the population with higher SES, since the ability to access resources and healthcare may be quite different.

Additionally, SES may have also affected some other determinants, such as the frequency of attending ANC or the presence and use of pregnancy supplements that may have influenced maternal and perinatal outcomes. Future work should strive to include participants from a wider socioeconomic distribution in order to help understand these relationships better and to enhance generalizability.

Finally, we stress that large sample sizes are needed to develop comprehensive solutions, as it is essential to acknowledge small, recruited samples in some subgroups, such as the participants from the Al Karak district and adolescents, despite their reported high rates of maternal anemia.

## 5. Conclusions

Our study showed a dramatic drop in the prevalence of maternal anemia in southern Jordan, from 56.5% in 2019 to 36.8%. However, it remains higher than the 2019 global average and falls within the WHO-defined range for a moderate public health problem. Several independent risk factors and modifiable risk factors for anemia have been identified, with ANC attendance, and folic acid and multivitamin supplementation being those that can be directly addressed by the health sector.

In contrast, education and socioeconomic status lie beyond the immediate scope of healthcare interventions and require broader, multi-sectoral efforts.

Targeted interventions to this problem should therefore encourage early ANC attendance, particularly among high-risk groups, including women with low income and low levels of education and those in younger age groups. Early ANC attendance would allow for the timely iron and folic acid supplementation, dietary counseling, nutritional campaigns on pregnancy needs, and the promotion of affordable and accessible alternatives, thereby reducing anemia its general prevalence.

For adolescent pregnancy, where levels of anemia are provisionally classified as severe by WHO standards, more national efforts should be made towards preventing early marriage and delaying childbearing in order to address this demographic’s high vulnerability.

## Figures and Tables

**Table 1:** Sociodemographic, medical, and diet-related characteristics of respondents with prevalence, degree of anemia, and associations, N = 474.

Variable	Frequency	Median (Mean ± SD ^1^)	Hemoglobin Level (gm/dL) 11.6 (11.42 ± 1.43)				*p*-Value
			<7 Severe	7–9.9 Moderate	10–10.9 Mild	>11 Normal	
Age		30 (29.09 ± 6.34)					
<20	25 (5.3)		0	4 (16)	8 (32)	13 (52)	0.910
20–25	120 (25.3)			17 (14.2)	24 (20)	79 (65.8)	
26–34	225 (47.5)			31 (13.8)	50 (22.2)	144 (64)	
35–45	104 (21.9)			17(16.4)	23(22.1)	64 (61.5)	
BMI ^2^ (kg/m^2^)		24.9 (26.32 ± 6.24)					
<18.5	47 (9.9)		0	7 (14.9)	13 (27.7)	27 (57.4)	0.348
18.5–24.9	191 (40.3)			32 (16.8)	43 (22.5)	116 (60.7)	
25–29.9	134 (28.3)			19 (14.1)	25 (18.7)	90 (67.2)	
30–34.9	34 (7.2)			6 (17.6)	9 (26.5)	19 (55.9)	
≥35	68 (14.3)			5 (7.3)	15 (22.1)	48 (70.6)	
District							
Al Karak	385 (81.2)		0	15 (3.9)	119 (30.9)	251 (65.2)	0.040 *
Al Qatranah	21 (4.4)			2 (9.5)	8 (38.1)	11 (52.4)	
Ghor Al Safi	39 (8.2)			2 (5.1)	22 (56.4)	15 (38.5)	
Al Tafilah	29 (6.2)			2 (6.9)	7 (24.1)	20 (69.0)	
Education							
Elementary	92 (19.4)		0	11 (12)	42 (45.7)	39 (42.4)	<0.001 *
Secondary	192 (40.5)			7 (3.7)	69 (36.3)	114 (60)	
University	190 (40.1)			2 (1)	43 (22.4)	147 (76.6)	
Employment							
No	377 (79.5)		0	230 (61)	128 (34)	19 (5)	0.042 *
Yes	97 (20.5)			70 (72.2)	26 (26.8)	1 (1)	
Household income/JOD ^3^		350 (373.21 ± 216.40)					
<200	150 (31.6)		0	36 (24)	47 (31.3)	67 (44.7)	<0.001 *
200–500	250 (52.7)			29 (11.6)	44 (17.6)	177 (70.8)	
501–1000	70 (14.8)			4 (5.7)	14 (20)	52 (74.3)	
>1000	4 (0.8)			0	0	4 (100)	
Family size		3 (4.05 ± 2.12)					
Two	142 (30)		0	18 (12.7)	26 (18.3)	98 (69)	0.223
Three	100 (21.1)			15 (15)	20 (20)	65 (65)	
Four	84 (17.7)			15 (17.9)	17 (20.2)	52 (61.9)	
≥Five	148 (31.2)			21 (14.2)	42 (28.4)	85 (57.4)	
Daily tea intake							
None	234 (49.4)		0	39 (16.7)	49 (20.9)	146 (62.4)	0.267
Once	139 (29.3)			15 (10.8)	29 (20.9)	95 (68.3)	
Multiple	101 (21.3)			15 (14.9)	27 (26.7)	59 (58.4)	
Meals/day							
Once	54 (11.4)		0	22 (40.7)	11 (20.4)	21 (38.9)	0.001 *
Twice	191 (40.3)			22 (11.5)	39 (20.4)	130 (68.1)	
3 times	187 (39.5)			19 (10.2)	48 (25.7)	120 (64.1)	
≥4 times	42 (8.9)			6 (14.3)	7 (16.7)	29 (69)	
Meat intake							
Daily	17 (3.6)		0	1 (5.9)	4 (23.5)	12 (70.6)	<0.001 *
Twice/week	75 (15.8)			3 (4)	14 (18.7)	58 (77.3)	
Once/week	161 (34)			15 (9.3)	29 (18)	117 (72.7)	
1–3 times/month	221 (46.6)			50 (22.6)	58 (26.2)	113 (51.1)	
Smoking							
Active							
No	457 (96.4)		0	20 (4.4)	148 (32.4)	289 (63.2)	0.561
Yes	17 (3.6)			1 (5.8)	5 (29.4)	11 (64.7)	
Passive							
No	138 (29.1)		0	6 (4.3)	38 (27.5)	94 (68.1)	0.174
Yes	336 (70.9)			14 (4.2)	116 (34.5)	206 (61.3)	
Medical Comorbidities							
No	441 (93)		0	61 (13.8)	100 (22.7)	280 (63.5)	0.852
Yes	33 (7.0)			8 (24.2)	5 (15.2)	20 (60.6)	
Hemorrhoids							
No	422 (89)		0	60 (14.2)	96 (22.8)	266 (63)	0.879
Yes	52 (11)			9 (13.3)	9 (13.3)	34 (65.4)	
Peptic ulcer							
No	439 (92.6)		0	73 (16.6)	85 (19.4)	281 (64)	0.276
Yes	35 (7.4)			6 (17.1)	10 (28.6)	19 (54.3)	

^1^ SD, standard deviation; ^2^ BMI, body mass index; ^3^ JOD, Jordanian dinar = 1.52 US dollars. * Statistically significant, *p*-value of ≤0.05.

**Table 2:** Obstetric characteristics of respondents with the prevalence, degree of anemia, and associations, N = 474.

Variable	Frequency	Median (Mean ± SD ^1^)	Hemoglobin Level (gm/dL) 11.6 (11.42 ± 1.43)				*p*-Value
			<7 Severe	7–9.9 Moderate, n (%)	10–10.9 Mild, n (%)	>11 Normal, n (%)	
Gestational age		28 (25.24 ± 7.87)					
First trimester	60 (12.7)		0	2 (3.3)	17 (28.3)	41 (68.3)	0.163
Second trimester	173 (36.5)			12 (6.9)	44 (25.4)	117 (67.6)	
Third trimester	241 (50.8)			6 (2.5)	93 (38.6)	142 (58.9)	
Total	474			20	154	300	
Parity		1 (2.03 ± 2.10)					
Primigravida	143 (30.1)		0	18 (12.6)	27 (18.9)	98 (68.5)	0.278
1–4	253 (53.4)			43 (17)	57 (22.5)	153 (60.5)	
≥5	78 (16.5)			8 (10.3)	21 (26.9)	49 (62.8)	
Total	474			69	105	300	
Interpregnancy interval	n (% Multiparous = 331)						
<1 year	118 (35.6)		0	18 (15.2)	37 (31.4)	63 (53.4)	0.159
1–2 years	80 (24.2)			12 (15)	16 (20)	52 (65)	
>2 years	133 (40.2)			21 (15.8)	26 (19.5)	86 (64.7)	
Total	331			51	79	201	
Antenatal care							
Regular	356 (75.1)		0	9 (2.5)	101 (28.4)	246 (69.1)	<0.001 *
Irregular	118 (24.9)			11 (9.3)	53 (44.9)	54 (45.8)	
Total	474			20	154	300	
Previous C/S ^2^	n (% Multiparous = 331)						
No	126 (38.1)		0	20 (15.9)	30 (23.8)	76 (60.3)	0.213
Yes	205 (61.9)			35 (17.1)	48 (23.4)	122 (59.5)	
Total	331			55	78	198	
Folic acid intake							
No	29 (6.1)		0	6 (20.7)	15 (51.7)	8 (27.6)	<0.001 *
Yes	445 (93.9)			14 (3.1)	139 (31.2)	292 (65.6)	
Total	474			20	154	300	
Iron supplement							
No	170 (35.9)		0	15 (8.8)	60 (35.3)	95 (55.9)	0.010 *
Yes	304 (64.1)			5 (1.6)	94 (30.9)	205 (67.4)	
Total	474			20	154	300	
Vitamin supplements							
No	180 (38)		0	14 (7.8)	80 (44.4)	86 (47.8)	<0.001 *
Yes	294 (62)			6 (2.0)	74 (25.2)	214 (72.8)	
Total	474			20	154	300	

^1^ SD, standard deviation; ^2^ C/S, cesarean section. * Statistically significant, *p*-value of ≤0.05.

**Table 3:** Sociodemographic, economic, medical, and diet-related predictors of anemia among the respondents.

Variable	Categories	AOR ^1^ (95% CI ^2^)	*p*-Value
District	Al Karak	1	
	Al Qatranah	1.289 (0.666, 2.496)	0.531
	Ghor Al Safi	1.722 (1.244, 2.384)	0.014 *
	Al Tafilah	0.788 (0.370, 1.678)	0.619
Education	Elementary	1	
	Secondary	0.694 (0.542, 0.889)	0.007 *
	University	0.407 (0.298, 0.555)	<0.001 *
Employment	Yes	0.603 (0.370, 0.985)	0.045 *
	No	1	
Household income/JOD ^3^	<200	1	
	200–500	0.333 (0.218, 0.508)	<0.001 *
	501–1000	0.279 (0.150, 0.522)	<0.001 *
	>1000	No anemia cases	No anemia cases
Meals/day	Once	1	
	Twice	0.299 (0.160, 0.558)	<0.001 *
	3 times	0.355 (0.190, 0.663)	0.002 *
	≥4 times	0.285 (0.122, 0.669)	0.004 *
Meat intake	Daily	0.436 (0.149, 1.279)	0.138
	Twice/week	0.307 (0.168, 0.560)	<0.001 *
	Once/week	0.393 (0.255, 0.608)	<0.001 *
	1–3 times/month	1	

^1^ AOR: adjusted odd ratio; ^2^ CI: confidence interval; ^3^ JOD, Jordanian dinar = 1.52 US dollars. * Statistically significant, *p*-value of ≤0.05.

**Table 4:** Obstetric predictors of anemia among the respondents.

Variable	Categories	AOR ^1^ (95% CI ^2^)	*p*-Value
Antenatal care	Regular	0.457 (0.293, 0.715)	0.001 *
	Irregular	1	
Folic acid intake	Yes	0.327 (0.135, 0.794)	0.014 *
	No	1	
Iron supplement	Yes	1.230 (0.749, 2.021)	0.413 *
	No	1	
Vitamin supplement	Yes	0.382 (0.235, 0.621)	<0.001 *
	No	1	

^1^ AOR: adjusted odd ratio; ^2^ CI: confidence interval; * Statistically significant, *p*-value of ≤0.05.

**Table 5:** Sociodemographic, diet-related, and obstetric characteristics of the adolescent respondents with prevalence and degree of anemia, N = 25.

Variable	Frequency (%)	Hemoglobin Level (gm/dL)			
		<7 Severe	7–9.9 Moderate, n (%)	10–10.9 Mild, n (%)	>11 Normal, n (%)
BMI ^1^ (kg/m^2^)					
<18.5	5 (20)	0	1 (20)	3 (60)	1 (20)
18.5–24.9	11 (44)		1 (9.1)	3 (27.3)	7 (63.6)
25–29.9	7 (28)		1 (14.3)	1 (14.3)	5 (71.4)
30–34.9	2 (8)		0	1 (50)	1 (50)
District					
Karak	13 (52)	0	2 (15.4)	5 (38.5)	6 (46.2)
Al Qatranah	4 (16)		1 (25)	1 (25)	2 (50)
Ghor Al Safi	6 (24)		2 (33.3)	2 (33.3)	2 (33.3)
Tafilah	2 (8)		0	1 (50)	1 (50)
Education					
Elementary	15 (60)	0	2 (13.3)	7 (46.7)	6 (40)
Secondary	10 (40)		2 (20)	2 (20)	6 (60)
Employment					
No	25 (100)	0	4 (16)	8 (32)	13 (52)
Household income/JOD ^2^					
<200	11 (44)	0	2 (18.2)	5 (45.5)	4 (36.3)
200–500	14 (56)		1 (7.1)	4 (28.6)	9 (64.3)
Family size					
Two	17 (68)	0	3 (17.7)	4 (23.5)	10 (58.8)
Three	5 (20)		0	4 (80)	1 (20)
Four	3 (12)		1 (33.3)	1 (33.3)	1 (33.3)
Daily tea intake					
None	8 (32)	0	1 (12.5)	3 (37.5)	4 (50)
Once	12 (48)		1 (8.3)	4 (33.3)	7 (58.4)
multiple	5 (20)		1 (20)	2 (40)	2 (40)
Meals/day					
Once	4 (16)	0	0	3 (75)	1 (25)
Twice	6 (24)		0	1 (16.7)	5 (83.3)
3 times	13 (52)		2 (15.3)	6 (46.2)	5 (38.5)
≥4 times	2 (8)		1 (50)	0	1 (50)
Meat intake					
Once/week	10 (40)	0	1 (10)	3 (30)	6 (60)
1–3 times/month	15 (60)		2 (13.3)	6 (40)	7 (46.7)
Smoking					
Active					
No	23 (92)	0	4 (17.4)	8 (34.8)	11 (47.8)
Yes	2 (8)		0	0	2 (100)
Passive					
No	7 (28)	0	1 (14.3)	2 (28.6)	4 (57.1)
Yes	18 (72)		3 (16.7)	6 (33.3)	9 (50)
Gestational age					
Second trimester	9 (36)	0	0	5 (55.6)	4 (44.4)
Third trimester	16 (64)		4 (25)	4 (25)	8 (50)
Parity					
Primigravida	17 (68)	0	3 (17.7)	4 (23.5)	10 (58.8)
1–4	8 (32)		0	5 (62.5)	3 (37.5)
Interpregnancy interval	n (% Multiparous)				
<1 year	5 (62.5)	0	0	4 (80)	1 (20)
1–2 years	3 (37.5)		0	1 (33.3)	2 (66.7)
Antenatal care					
Regular	14 (56)	0	1 (7.1)	5 (35.7)	8 (57.2)
Irregular	11 (44)		3 (27.2)	4 (36.4)	4 (36.4)
Previous C/S ^3^	n (% Multiparous)				
No	4 (50)	0	0	1 (25)	3 (75)
Yes	4 (50)		0	4 (100)	0
Folic acid intake					
No	5 (20)	0	0	3 (60)	2 (40)
Yes	20 (80)		3 (15)	6 (30)	11 (55)
Iron supplements					
No	9 (36)	0	1 (11.1)	3 (33.3)	5 (55.6)
Yes	16 (64)		2 (12.5)	6 (37.5)	8 (50)
Vitamin supplements					
No	12 (48)	0	2 (16.6)	5 (41.7)	5 (41.7)
Yes	13 (52)		1 (7.7)	4 (30.8)	8 (61.5)

^1^ BMI, body mass index; ^2^ JOD, Jordanian dinar = 1.52 US dollars; ^3^ C/S: cesarean section.

## Data Availability

The original contributions presented in the study are included in the article, further inquiries can be directed to the corresponding author.
